# Combination of Hydrolysable Tannins and Zinc Oxide on Enterocyte Functionality: In Vitro Insights

**DOI:** 10.3390/biom14060666

**Published:** 2024-06-06

**Authors:** Francesca Ciaramellano, Lucia Scipioni, Benedetta Belà, Giulia Pignataro, Giacomo Giacovazzo, Clotilde Beatrice Angelucci, Roberto Giacominelli-Stuffler, Alessandro Gramenzi, Sergio Oddi

**Affiliations:** 1 Department of Veterinary Medicine, University of Teramo, 64100 Teramo, Italygpignataro@unite.it (G.P.);; 2 European Center for Brain Research (CERC), Santa Lucia Foundation IRCCS, 00143 Rome, Italy; lucia.scipioni@graduate.univaq.it; 3 Department of Biotechnological and Applied Clinical Sciences, University of L’Aquila, Via Vetoio Snc, 67100 L’Aquila, Italy

**Keywords:** Caco-2 cells, epithelial barrier function, gastrointestinal diseases, zinc oxide (ZnO), hydrolysable tannins (HTs), epithelial barrier function, oxidative stress

## Abstract

The management of gastrointestinal disease in animals represents a significant challenge in veterinary and zootechnic practice. Traditionally, acute symptoms have been treated with antibiotics and high doses of zinc oxide (ZnO). However, concerns have been raised regarding the potential for microbial resistance and ecological detriment due to the excessive application of this compound. These concerns highlight the urgency of minimizing the use of ZnO and exploring sustainable nutritional solutions. Hydrolysable tannins (HTs), which are known for their role in traditional medicine for acute gastrointestinal issues, have emerged as a promising alternative. This study examined the combined effect of food-grade HTs and subtherapeutic ZnO concentration on relevant biological functions of Caco-2 cells, a widely used model of the intestinal epithelial barrier. We found that, when used together, ZnO and HTs (ZnO/HTs) enhanced tissue repair and improved epithelial barrier function, normalizing the expression and functional organization of tight junction proteins. Finally, the ZnO/HTs combination strengthened enterocytes’ defense against oxidative stress induced by inflammation stimuli. In conclusion, combining ZnO and HTs may offer a suitable and practical approach for decreasing ZnO levels in veterinary nutritional applications.

## 1. Introduction 

The intestinal epithelium serves as a protective shield against harmful substances and microorganisms that may enter the gut. This protective barrier mainly consists of a single layer of epithelial cells known as enterocytes, which are tightly interconnected through protein structures called tight junctions [1]. This physical barrier prevents the uncontrolled passage of microbes, toxins, and undigested food particles from the gut into the bloodstream [2]. Various factors, such as environmental conditions, pathophysiological changes, and infections, can compromise the structural integrity of this protective barrier, leading to intestinal disorders [3,4]. During the inflammatory processes associated with these heterogeneous conditions, enterocytes react to cytokines released by immune cells, modulating processes like proliferation, migration, antioxidative responses, and intercellular adhesions [5,6].

Zinc oxide (ZnO) is an inorganic compound employed in veterinary medicine, particularly for treating intestinal inflammation and diarrhoea [7]. It is generally well tolerated and effective at restoring the balance of zinc in the gut, which helps to manage these conditions [8]. There are many properties ascribed to the usefulness of ZnO in intestinal pathologies, the most credited among them being its bacteriostatic and bactericidal activities that shape the intestinal microbiome [9,10]. The research carried out has shown that ZnO can preserve the integrity and function of epithelial barriers and might enhance the process of mucosal repair and paracellular permeability [11]. The activation of the PI3K/Akt/mTOR signalling pathway by inorganic zinc sources plays a role in improving intestinal barrier function by enhancing cell differentiation and the expression of the tight junction protein zonula occludens-1 [12] However, prolonged use of ZnO can contribute to antibiotic resistance and environmental pollution [7,13,14]. Indeed, recent investigations showed that ZnO treatment of post-weaning pigs can favour the selection and development of antimicrobial-resistant (AMR) and multidrug-resistant (MDR) *E. coli* [15]. Furthermore, bacterial in vitro studies provided evidence that stress conditions such as heavy metals exposure may play an important role in potentially inducing acquisition of antibiotic resistance genes through horizontal gene transfer [16,17]. To address these concerns, it is crucial to explore more sustainable and safe alternatives for treating gastrointestinal issues in animals, including better livestock management, judicious antibiotic use, and the use of natural alternatives like probiotics, prebiotics, and plant extracts.

In this context, hydrolysable tannins (HTs) have gained attention as a potential nutraceutical approach for managing intestinal pathologies [18,19]. These natural polyphenolic compounds are extracted from diverse plant sources and have a long history in folk medicine for treating gastrointestinal issues. As polyphenols, HTs have proven antioxidant activity; moreover, some detailed in vitro studies have described trophic effects on enterocytes derived from different animal species [20,21,22].

During digestive processes, both ZnO and HTs undergo significant modifications. Zinc oxide may undergo dissolution and subsequent complexation with various organic compounds present in the digestive tract [23,24]. Hydrolysable tannins, on the other hand, are susceptible to enzymatic hydrolysis, resulting in the release of phenolic acids and smaller polyphenolic compounds. The interaction between ZnO and HTs can lead to the formation of complexes due to the coordination of hydroxyl groups present in the tannins with zinc ions [25,26,27,28]. These complexes may exhibit altered chemical properties compared to the individual components. Furthermore, the acidic nature of HTs can influence the local pH in the digestive tract, potentially affecting the solubility and reactivity of zinc oxide [24,29]. Additionally, the presence of other dietary components, such as proteins and carbohydrates, can further modulate the interactions between ZnO and HTs [29]. These complex interactions highlight the dynamic nature of digestive processes and the potential for synergistic or antagonistic effects between dietary constituents. 

This research specifically delves into the possible combinatorial effect of food-grade HTs and reduced ZnO concentrations on the vital biological functions exhibited by Caco-2 cells, a widely used model for studying the intestinal epithelial barrier. Our findings demonstrate that the combined application of ZnO and HTs (referred to as ZnO/HTs) could enhance cell proliferation and tissue repair processes and improve the performance of the epithelial barrier, in particular by enhancing tight junction proteins. Furthermore, the ZnO/HTs blend reinforced the enterocytes’ resilience against the oxidative stress provoked by inflammatory agents. In summary, the strategic pairing of ZnO with HTs emerges as a promising solution for minimizing ZnO concentrations in animal feed supplements.

## 2. Materials and Methods

### 2.1. Reagents

The chemicals used were of the purest analytical grade. Dulbecco’s modified Eagle’s medium (DMEM), foetal bovine serum (FBS), and other cell culture reagents were purchased from Corning (Corning, New York, NY, USA). IFN-γ and M-CSF were purchased from Miltenyi Biotec (Bergisch Gladbach, Germany). Ficoll-Hypaque was obtained from Pharmacia (Uppsala, Sweden), while the nonessential amino acids (NEAA) and CellROX Green Reagent were from Invitrogen (Carlsbad, CA, USA). All other chemicals were purchased from Sigma-Aldrich (St. Louis, MO, USA), unless stated otherwise.

### 2.2. Preparation of Compounds 

Zinc oxide (CAS 1314-13-2, Sigma-Aldrich) was prepared in stock solutions in 5% (*v*/*v*) acetic acid in double-distilled H_2_O. Different batches of Chestnut wood (*Castanea sativa* Mill.)-derived HTs in the food-grade formulation were characterized through qualitative–quantitative analysis (as summarized in Table 1) and dissolved in double-distilled H_2_O. After solubilization, the compounds were sterile-filtered, aliquoted, and stored at −20 °C until use.

### 2.3. Cell Culture

The Caco-2 cell line, derived from a human colon adenocarcinoma (ATCC, Rockville, MD, USA), was used between passages 15 and 35. Cells were routinely maintained in DMEM supplemented with 4.5 g/L glucose, 10% (*v*/*v*) heat-inactivated FBS, 1% (*v*/*v*) L-glutamine, and 1% (*v*/*v*) NEAA at 37 °C in a 5% CO_2_-humidified atmosphere.

### 2.4. Viability and Proliferation Assay

Cell viability was determined by 3-(4,5-dimethylthiazol-2-yl)-2,5-diphenyl tetrazolium bromide (MTT) assay as previously described [30]. Briefly, Caco-2 cells were seeded in 96-well culture plates (1 × 10^4^ cells/well) in a complete culture medium and then incubated for 24 h. Next, cells were treated for 24 or 48 h with different HTs concentrations (15, 40, 100, 200, 400, and 600 μg/mL) alone or in the presence of 0.8 μg/mL ZnO. The medium was removed, and cells were incubated with 5 mg/mL of MTT reagent dissolved in a complete medium. After 2 h, the supernatant was removed, and the formazan crystals were dissolved with 100 μL dimethyl sulfoxide. The optical densities were measured at 570 nm wavelength (microplate reader Varioskan Flash Spectral Scanning Multimode Reader; Thermo Scientific, Waltham, MA, USA) and the viability of cells was expressed as a percentage over control with the viability of non-treated control cells arbitrarily defined as 100%.

### 2.5. Monolayer Tip Scratch Test

For monolayer tip scratch test experiments, cells were seeded in a 12-well plate (1 × 10^5^ cells/well). After reaching 100% confluence, cell monolayers were scratched using a 10 µL sterile pipette tip [31]. Cells were then rinsed with PBS and treated with ZnO (0.8 μg/mL), HTs (40 μg/mL), or the combination of the substances (ZnO/HTs) in a complete medium. Scratch closure was recorded at 0, 24, and 48 h using a digital microscope (PAULA, Personal AUtomated Lab Assistant; LEICA Microsystems, Wetzlar, Germany). Scratch widths were measured using digital imaging system software (Leica Application Suite V4.2).

### 2.6. PBMCs Isolation

Peripheral blood mononuclear cells (PBMCs) were isolated from venous blood samples of healthy donors using a density gradient on Ficoll-Hypaque according to standard procedures [32]. In detail, 10 mL of peripheral blood was diluted 1:2 with sterile PBS and gently stratified on top of 15 mL of Ficoll-Hypaque and centrifuged for 30 min at 100× *g* at 20 °C without use of the brake. The PBMCs contained in the interphase formed between Ficoll and plasma were gently collected with a sterile Pasteur pipette and washed twice in sterile PBS for 10 min and resuspended in RPMI 1640 complete medium supplemented with 10% (*v*/*v*) of inactivated human serum.

### 2.7. Isolation of Monocytes, Differentiation into Macrophages and M1 Polarization

Monocytes were isolated by adhesion in a complete RPMI 1640 medium for two hours. Subsequently, non-adherent cells were removed, and adherent monocytes were gently washed with PBS and cultured in fresh complete medium, supplemented with 50 ng/mL M-CSF, to induce differentiation into M0 (homeostatic macrophages) for 6 consecutive days, with a complete renewal of the medium on days 2 and 4 [33]. On day 6, cells were harvested with a trypsin-EDTA solution and plated in 24-well plates for stimulation at a density of 2 × 10^5^ cells/well. Polarization into M1 (pro-inflammatory macrophages) was achieved in the presence of 100 ng/mL LPS from *Escherichia coli* O111:B4 and 10 ng/mL IFN-γ.

### 2.8. Macrophage-Conditioned Medium

For the oxidative stress assay, immunofluorescence, and qPCR studies, we simulated the inflammatory process, taking advantage of the macrophages’ ability to release a specific pool of cytokines into the culture medium. In brief, fully differentiated macrophages were stimulated for 24 h to induce M1 polarization, as indicated in Section 2.7. Under this stimulus, macrophages released a massive amount of prototypical pro-inflammatory cytokines and soluble factors such as MCP1, eotaxin, eotaxin-3, IL12p70, IL-1α, IL15, TNF-β, IL-6, TNF-α, IL12p40, IL-13, and IL-2 [34] into the conditioned culture medium (CDM). Following this, the medium was refreshed, and macrophages were allowed to continue producing cytokines for an additional 24 h. Subsequently, the medium, defined in this passage as CDM, was harvested, centrifuged, and used immediately or stored at −80 °C.

### 2.9. Oxidative Stress Assay

In order to detect reactive oxygen species (ROS), Caco-2 cells were seeded at a density of 10,000 cells/well in black plates. After 24 h, cells were challenged with CDM alone or in combination with ZnO (0.8 μg/mL), HTs (40 μg/mL), or a combination of the substances (ZnO/HTs). Oxidative stress was monitored using CellROX Green Reagent according to the manufacturer’s instructions. Nuclei were counterstained with Hoechst 33342. After 1 h, cells were washed with PBS and imaged using a digital microscope (ZOE Fluorescent Cell Imager, Bio-Rad, Hercules, CA, USA). The data were exported as TIFF files and analysed using Fiji software (National Institutes of Health; version 2.3.0/1.53f, released on 13 September 2021; https://imagej.net/Fiji).

### 2.10. Macrophages and Caco-2 Cocultures and TEER Measurement

For the cocultures, 80 × 10^3^ Caco-2 cells were seeded in transwells with a surface area of 33.6 mm^2^ (Greiner bio-one Thin Certs TC Inserts, 0.4 µM pore size) and cultured in complete medium for 21 days to obtain fully differentiated cells [35]. To ensure monolayer integrity, transepithelial electrical resistance (TEER) values were monitored using a Millicell ERS meter (Millipore, Bedford, MA, USA). Only monolayers with more than 600 Ω·cm^2^ of TEER, which guaranteed their integrity and differentiation, were selected for coculture experiments. The transwells were then transferred to a 24-well plate with M1 macrophages (200,000 cells/well) obtained as previously described. HTs, ZnO, and ZnO/HTs were added to the apical chamber at chosen concentrations. TEER values were measured at the time of coculture establishment and after 48 h and expressed as a percentage of the initial value, calculated as (TEER at 48 h from coculture establishment/TEER at the coculture establishment) × 100.

### 2.11. Confocal Microscopy

Caco-2 cells were cultured on glass coverslips until fully differentiated, then challenged with 200 µL of CDM alone or in combination with ZnO/HTs for 48 h, followed by rinsing with PBS. The cells were fixed in a solution of 3% paraformaldehyde and 4% sucrose for 30 min, permeabilized with 0.1% Triton X-100 for 15 min, blocked in 5% BSA for 1 h at room temperature, and then rinsed again with PBS. Staining with occludin monoclonal antibody (1:250; OC-3F10, Invitrogen) or ZO-1 monoclonal antibody (1:150; ZO1-1A12, Invitrogen) was performed overnight at 4 °C in a dark, humid chamber. Monolayers were washed twice for 5 min each with PBS and then incubated for 1 h at room temperature with anti-mouse Alexa Fluor 488-labeled secondary antibody (Thermo Fisher Scientific). During the final step, the cells were incubated with DAPI for counterstaining and then mounted with ProLong Gold Antifade Mountant (Thermo Fisher Scientific). Samples were examined using a confocal fluorescence microscope (LSM 510; Zeiss, Leipzig, Germany). For image analysis, data from high-resolution images of 11/12 cells from 3 independent experiments were acquired for each sample.

### 2.12. RNA Preparation and Real-Time RT-PCR Analysis 

For the mRNA extraction procedure, the ReliaPrep RNA Cell Miniprep System (Promega) was utilized following the provided instructions. The extracted mRNA was quantified by measuring absorbance at 260 nm using a NanoDrop UV–Vis spectrophotometer (Thermo Fisher), and cDNA synthesis was carried out with the SensiFAST cDNA Synthesis Kit (Bioline). The resulting cDNAs were analysed in qPCR using Taqman probes (Applied Biosystems, Life Technologies, Carlsbad, CA, USA). The gene names and their assay identification numbers were as follows: ACTB Hs01060665 g1, MMP9 Hs00957562 m1. The assays were conducted using a StepOne Real-Time PCR System sequence detector (Applied Biosystems, Life Technologies, Carlsbad, CA, USA). The results were normalized by calculating the ΔC_t_, where ΔC_t_ = C_t_(Housekeeping gene) − C_t_(Target gene), and data are expressed as 2^−ΔCt^. 

### 2.13. Statistical Analysis

The data underwent evaluation for both distributional characteristics and adherence to normality utilizing the Shapiro–Wilk test. Depending on the outcome, either parametric or non-parametric tests were employed for subsequent statistical analyses. The statistical test used for each analysis is specified in the caption beneath the corresponding graph. All results are reported as mean ± standard deviation (S.D.). *p* < 0.05 was chosen to establish significance. Data were elaborated and analysed statistically using the R Statistical Package (R version 4.2.2 (31 October 2022)) within RStudio software (2022.12.0 + 353 version: https://rstudio.com/) or the GraphPad Prism (version 9). The statistical methods used for each analysis are specified in the figure legends.

## 3. Results

### 3.1. ZnO Exerted a Protective Effect against the Cytotoxicity of HTs When Co-Administered to Caco-2 Cells 

Firstly, we evaluated the effects on cell viability and proliferation of increasing doses of HTs and ZnO, administered alone or in combination, at two different time points (i.e., 24 h and 48 h). ZnO was found to be non-toxic up to the chosen maximum concentration of 8 μg/mL, corresponding to 150 μM (Supplementary Figure S1). Based on previous animal study data, the ZnO concentration of 0.8 μg/mL (10 μM) was selected, which corresponds to a much lower value than those detected in the intestinal lumen of animals treated with ZnO at different therapeutic dosages which do not exert bactericidal activity [36,37,38,39]. HTs were found to be well tolerated by cells and began to have toxic effects at concentrations above 200 μg/mL. Notably, HT-induced toxicity was more evident after two days of treatment. 

**Figure 1:**
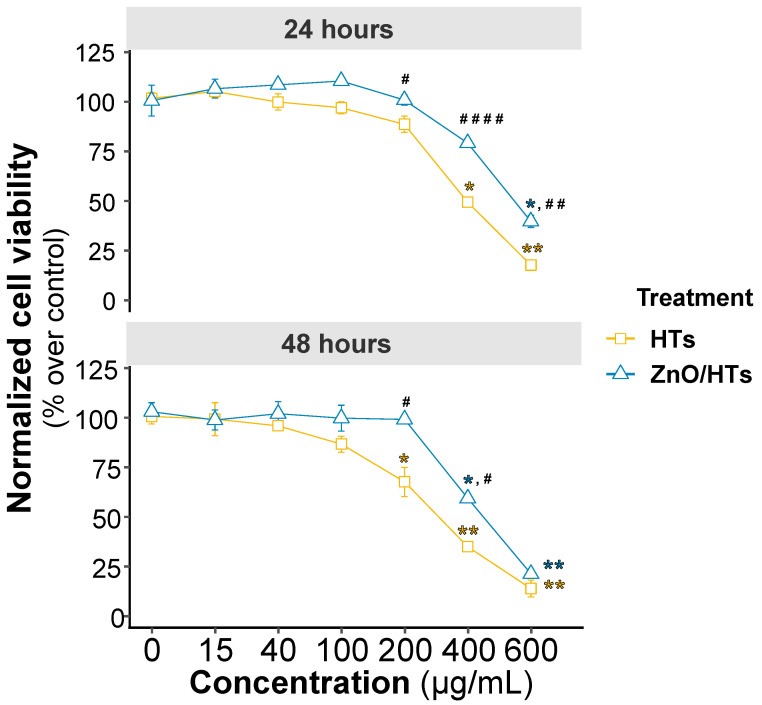
MTT assay for cytotoxicity and proliferation investigation. The diagrams represent the viability of Caco-2 treated with hydrolysable tannins (HTs) at different concentrations (15, 40, 100, 200, 400, and 600 μg/mL) and in combination with 0.8 μg/mL zinc oxide (ZnO/HTs) at two different exposure times: 24 and 48 h. The results of the MTT assay are expressed as the percentage of vitality of untreated cells and presented as the mean ± standard deviation of three independent experiments. Statistical differences were analysed by two-way analysis of variance, with the Bonferroni post hoc test. The notation of significance is indicated by an asterisk (*) when comparisons are made relative to untreated cells, whereas a hash mark (#) denotes the *p* value when contrasting the two treatments, HTs and ZnO/HTs, at each respective concentration. The levels of significance are indicated as follows: * *p* < 0.05, ** *p* < 0.01, all in comparison to the untreated control; ^#^ *p* < 0.05, ^##^ *p* < 0.01, ^####^ *p* < 0.0001, all in comparison to HTs.

To investigate the potential interaction between the two substances on cell viability, we tested all concentrations of HTs in the presence of 0.8 μg/mL of ZnO. Interestingly, we found that both at 24 and 48 h, the presence of ZnO significantly reduced the mortality of Caco-2 cells induced by high doses of HTs (Figure 1).

### 3.2. Combined ZnO/HTs Treatment Improved Scratch Closure Rates Compared to ZnO Alone

Based on cytotoxicity findings, a concentration of 40 μg/mL of HTs was selected in the presence of 0.8 μg/mL ZnO, a combination maintained throughout all further experiments. In order to investigate the potential effect of adding HTs to ZnO on the ability of cells to migrate during the healing process, confluent Caco-2 monolayers were subjected to a longitudinal incision, which was designed to mimic a lesion of the mucosa. The repopulation of the scratched areas was then monitored over time.

**Figure 2:**
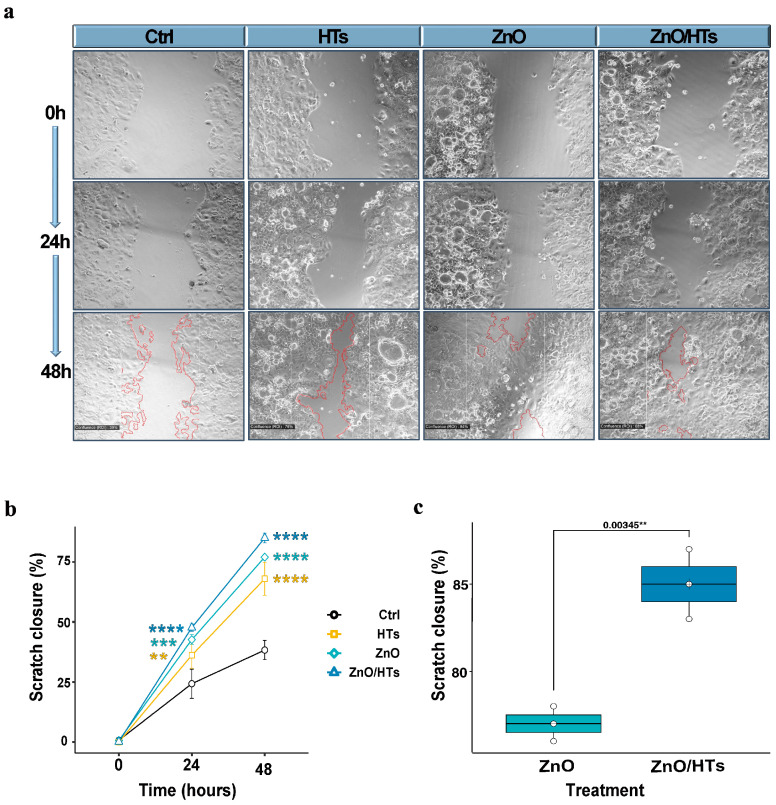
Assessment of monolayer tip scratch test in Caco-2 cells. (**a**) Representative images of the tip scratch assay are shown at 0 h, 24 h, and 48 h for both untreated cells (Ctrl) and those treated with zinc oxide (ZnO, 0.8 µg/mL), hydrolysable tannins (HTs, 40 µg/mL), or a combination of ZnO and HTs (ZnO/HTs). (**b**) The graph illustrates the progression of scratch closure over time by expressing the cell-covered surface area as a percentage after 24 and 48 h. The results are presented as the mean ± standard deviations from three independent experiments. (**c**) Comparison of the scratch closure efficacy of ZnO alone with its combined use with 40 µg/mL of HTs (ZnO/HTs). The box plots feature a central line indicating the median, a cross for the mean value, and top and bottom edges for the third and first quartiles, respectively. The “whiskers” show data within 1.5 × IQR (interquartile range), and white circles denote individual experimental data points. For part (**b**), a one-way ANOVA was employed, followed by multiple post hoc *t*-tests with Holm–Sidak’s correction for multiple comparisons. The significance levels are denoted as ** *p* < 0.01, *** *p* < 0.001, and **** *p* < 0.0001, all compared to the control. For panel (**c**), an unpaired *t*-test was used, with the *p* value from the statistical analysis presented above the line. ** *p* < 0.01.

The control group exhibited the slowest scratch closure rate, not reaching even 50% closure by 48 h. Used alone, ZnO accelerated scratch healing compared to the control, showing more than 40% and 70% closure by 24 and 48 h, respectively. Treatment with HTs also resulted in faster scratch closure than the control, mirroring the performance of the ZnO treatment. However, the combination treatment (ZnO/HTs) significantly enhanced scratch healing, achieving the highest percentage of scratch closure at both 24 and 48 h, well over 75% at the final time point (ZnO/HTs at 24 h: 47.7 ± 1.53%, ZnO/HTs at 48 h 85.0 ± 2%, *p* < 0.001; Figure 2c). Overall, the combination of ZnO and HTs appeared to offer a synergistic effect, providing the most effective treatment for scratch closure within the observed time frame.

### 3.3. Combined ZnO/HTs Treatment Attenuated Inflammatory-Induced Oxidative Stress

**Figure 3:**
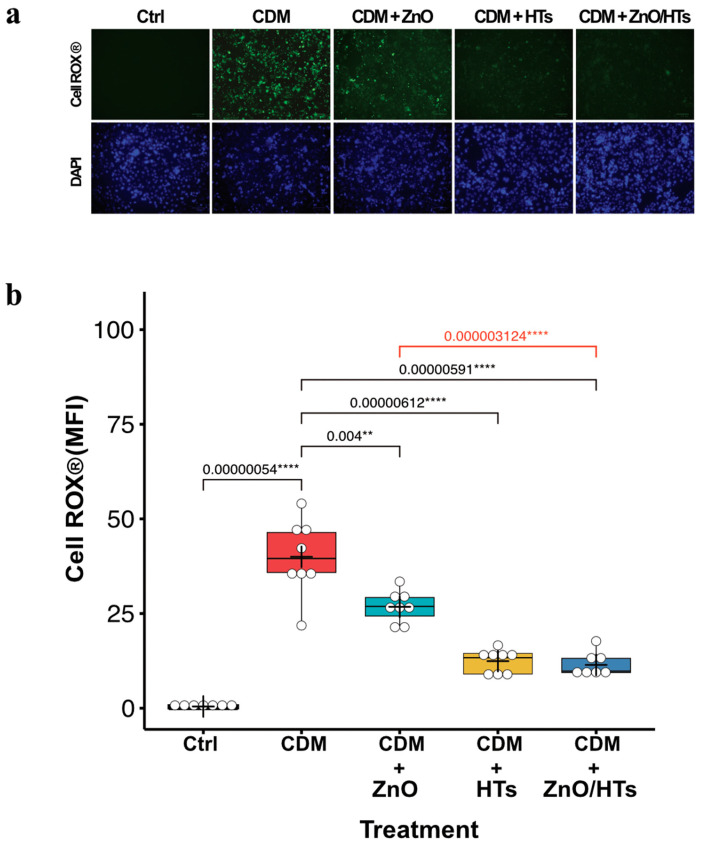
ZnO/HTs reduce ROS generation in inflammatory conditions. (**a**) Representative images of intracellular ROS detection with CellROX for untreated cells (Ctrl), cells treated with macrophage-conditioned medium (CDM) alone or in combination with zinc oxide (CDM + ZnO, 0.8 µg/mL), hydrolysable tannins (CDM + HTs, 40 µg/mL), ZnO and HTs (CDM + ZnO/HTs). Green = CellROX^®^ probe; Blue = DAPI staining of nuclei. (**b**) Probe quantification box plot obtained by calculating the mean fluorescence intensity (MFI) of Caco-2 nuclei. The box plots feature a central line indicating the median, a cross for the mean value, and top and bottom edges for the third and first quartiles, respectively. The “whiskers” show data within 1.5 × IQR (interquartile range), and white circles denote individual experimental data points. Statistical analysis was performed using a one-way ANOVA followed by post hoc multiple *t*-tests with Holm–Sidak’s correction for multiple comparisons. Significance levels are denoted as: ** *p* < 0.01 and **** *p* < 0.0001. CDM was derived from macrophages isolated from three distinct, healthy donors.

The generation of reactive oxygen species (ROS) is a crucial event in the development of numerous gastrointestinal inflammatory disorders [40]. To simulate inflammatory-induced oxidative stress, fully differentiated monolayers of Caco-2 cells were treated with a proinflammatory cytokine-rich medium obtained from isolated primary macrophages polarized towards a pro-inflammatory state (M1 macrophages; see Section 2). Exposure of Caco-2 cells to the conditioned medium (CDM) from M1 macrophage cultures induced a marked production of intracellular ROS revealed by using the redox-sensitive fluorogenic probe CellROX via fluorescence microscopy (Figure 3). 

As expected, ZnO and, to a major extent, HTs exhibited relevant antioxidant effects (Figure 3). Again, combining HTs with ZnO significantly reduced CDM-induced oxidative stress compared to use of ZnO alone (CDM + ZnO: 27 ± 4 MFI; CDM + ZnO/HTs: 11 ± 3 MFI; *p* < 0.0001; Figure 3b), further confirming the favourable effect of the two compounds.

### 3.4. Combined ZnO/HTs Treatment Fully Protected against Inflammatory-Induced Impairment in Epithelial Barrier Integrity

**Figure 4:**
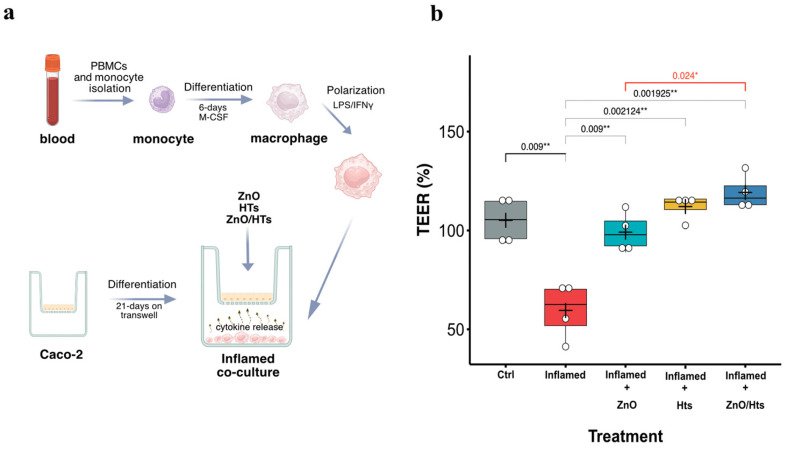
Enterocyte monolayer integrity in the coculture model of Caco-2 and human macrophages. (**a**) Schematic representation of coculture experiments between Caco-2 on transwell and human macrophages. (**b**) Percentage changes in transepithelial electrical resistance (TEER) values of Caco-2 cells untreated monoculture (Ctrl), cocultured with activated human macrophages (Inflamed), cocultured with activated human macrophages and treated with zinc oxide (ZnO, 0.8 µg/mL), hydrolysable tannins (HTs, 40 µg/mL), or a combination of ZnO and HTs (ZnO/HTs). TEER values are expressed as a percentage of the initial value, calculated as (TEER at 48 h from coculture establishment/TEER at the coculture establishment) × 100. The box plots feature a central line indicating the median, a cross for the mean value, and top and bottom edges for the third and first quartiles, respectively. The “whiskers” show data within 1.5 × IQR (interquartile range), and white circles denote individual experimental data points. Statistical analysis was performed using a one-way ANOVA followed by multiple post hoc *t*-tests with Holm–Sidak’s correction for multiple comparisons. Significance levels are denoted as: * *p* < 0.05, ** *p* < 0.01. Macrophages were isolated from *n* = 4 different healthy donors.

To further confirm the additive effect between ZnO and HTs in influencing the response of enteric monostratified epithelium to an inflammatory insult, these substances were tested in fully differentiated Caco-2 monolayers directly cocultured in a compartmentalized system with primary M1 macrophages (Figure 4a). 

Exposure of Caco-2 to soluble factors released in the culture media from M1 macrophages significantly decreased the barrier integrity, as measured by the reduction of % TEER values (Ctrl: 105 ± 12; inflamed: 73 ± 17%, *p* < 0.01). 

Interestingly, both ZnO and HTs treatments appeared to mitigate this functional impairment, each maintaining the mean % TEER back to near-baseline levels, suggesting a possible protective or restorative effect against the inflammatory insult. However, the most pronounced enhancement was observed combining ZnO with HTs treatment (inflamed + ZnO/HTs), which resulted in a significantly higher % TEER than the sole application of ZnO (Inflamed + ZnO: 99.12 ± 10.15%; Inflamed + ZnO/HTs: 119.2 ± 8.84%; *p* < 0.05). 

### 3.5. Combined ZnO/HTs Treatment Fully Prevented Alterations in the Expression/Distribution of Proteins Involved in Tight Junction Functionality

**Figure 5:**
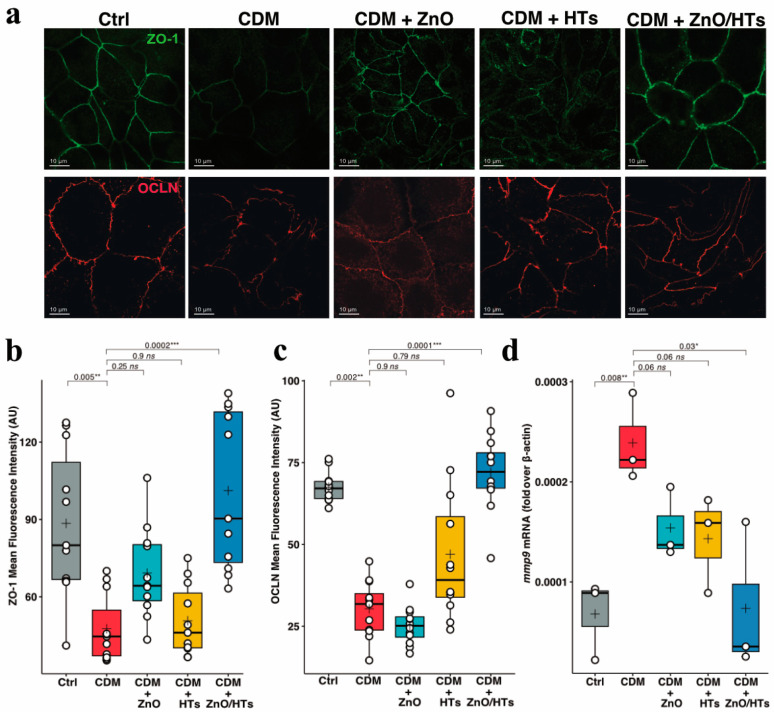
ZnO/HTs treatment preserves Caco-2 tight junction in the conditioned medium inflammatory model. (**a**) Immunofluorescence staining of the tight junction proteins zonula occludens-1(ZO-1) and occludin (OCLN) on Caco-2 untreated cells (Ctrl), on cells treated with M1 macrophage conditioned medium (CDM), and on cells treated with M1 macrophage conditioned medium and the combination of 0.8 µg/mL zinc oxide and 40 µg/mL hydrolysable tannins (CDM + ZnO/HTs). Red = occludin; Green = zonula occludens-1. (**b**,**c**) Quantification of the MFI of ZO-1 and OCLN immunostaining under each condition (as described in Section 2). Data are reported in box plots with a central line indicating the median, a cross for the mean value, and top and bottom edges for the third and first quartiles, respectively. The “whiskers” show data within 1.5 × IQR (interquartile range), and white circles denote individual experimental data points. Statistical analysis was conducted using the Kruskal–Wallis test to evaluate differences between groups (H = 16.98, *p* < 0.0002 for ZO-1; H = 23.37, *p* < 0.0001 for OCLN). Subsequently, a post hoc analysis was performed using Dunn’s test to identify specific differences between groups. (**d**) MMP9 gene expression in experiments with macrophage-conditioned medium. The results are expressed in relation to the mRNA of the *β*-actin constitutive gene. Significance is shown as a *p*-value, calculated using an unpaired *t*-test with the *p* value from the statistical analysis presented above the line. * *p* < 0.05, ** *p* < 0.01 and *** *p* < 0.001. The macrophage for the conditioned medium was obtained from *n* = 3 healthy donors.

Finally, we sought to evaluate the impact of ZnO/HTs treatment on the expression of relevant proteins involved in epithelial barrier functions, including zonula occludens-1 (ZO-1) and occludin (OCLN), as well as matrix metalloproteinase 9 (MMP9), an enzyme strongly associated with chronic enteritis [41]. In particular, we characterized the expression and subcellular distribution of ZO-1 and OCLN by confocal microscopy. As expected, fully differentiated Caco-2 cells were found to express high levels of both proteins with the typical localization at the cell-to-cell contacts. Exposure of Caco-2 monolayers to CDM resulted in a diffuse reduction in the fluorescent signal for both proteins, accompanied by discontinuous breaks occurring along the sites of the intercellular interaction. ZnO/HTs co-treatment fully prevented CDM-induced reorganization of ZO-1 and OCLN (Figure 5a–c).

Interestingly, CDM induced a robust increase in the MMP9 mRNA, which was 3.5-fold higher than the control group (Figure 5d). Our results showed that both substances induced a reduction in MMP9 gene expression compared to cells treated with CDM, but only the simultaneous treatment ZnO/HTs fully reverted this effect by bringing MMP9 mRNA expression closer to that of the control group.

## 4. Discussion

In the present study, we evaluated whether the combination of food-grade HTs with a reduced dosage of ZnO could enhance enterocytic functions in the context of mechanical and proinflammatory insults. Specifically, we used Caco-2 cells, a well-established model for studying intestinal barrier properties, and simulated physical and inflammatory damages on the integrity of fully differentiated cell monolayers. We found that adding HTs to a lower concentration of ZnO enhanced “healing” processes and improved the integrity of the epithelial monolayers by preserving tight junction proteins, possibly reducing the expression of MMP9 and potentiating the antioxidant defence within enterocytes.

Our research demonstrates that the incorporation of HTs to a subtherapeutical concentration of ZnO (ZnO/HTs) could significantly enhance the enterocytes’ capacity to respond to a physic lesion of the epithelial layer. The intestinal epithelium is crucial in forming a physical barrier against detrimental luminal substances, including bacteria, toxins, and antigens. Its rapid regenerative ability is essential in countering damage caused by immune system overactivation due to the infiltration of harmful substances into the intestine’s deeper layers. Our findings align with other studies, indicating the pro-healing effects of HTs in intestinal cells and animal models [21,22,42,43]. Both ZnO and HTs are capable of exerting pro-oxidant and antioxidant effects, depending on their concentrations and the presence of transition metals like Fe^3+^ and Cu^2+^, which are prevalent in biological systems [44,45,46,47]. In line with other studies, we observed that HTs, at a concentration of 40 µg/mL, exhibited a marked antioxidant action against ROS induced in Caco-2 cells by proinflammatory cytokines in the CDM [20]. Meanwhile, ZnO, administered at a suboptimal concentration of 10 µM, also displayed antioxidant activity, though it was less effective compared to HTs. These results suggest that within the selected concentration range, the addition of HTs to ZnO could compensate for the diminished antioxidant effect of ZnO when used at lower concentrations.

To mimic an inflammatory context, we employed an in vitro model involving compartmentalized culture, enabling interaction between the Caco-2 monostratified epithelium and activated M1 primary macrophages. In this model, proinflammatory factors released by activated macrophages impaired the integrity of the simulated epithelial barrier, as assessed by a marked reduction in the TEER values. Consistent with previous studies, various classes and formulations of HTs effectively mitigated the TEER reduction associated with cytokine activity [48,49]. Notably, our study revealed that the combined treatment of ZnO and HTs achieved higher TEER values, suggesting enhanced epithelial barrier integrity and reduced trans- and paracellular permeability. At the cellular level, paracellular permeability is regulated by intercellular junctions, including gap junctions, adherents junctions, desmosome junctions, and tight junctions, with tight junctions playing a critical role in maintaining cell polarity and preventing harmful substance translocation from the lumen to the bloodstream [50]

Our investigation found that the ZnO/HTs treatment preserved the cellular expression and localization of tight junction proteins ZO-1 and OCLN. This finding is consistent with various in vivo and in vitro studies concerning HTs, tannic acid, and other polyphenolic compounds [20,51,52]. Conversely, the existing literature on ZnO primarily focuses on nanoparticle formulations, which may induce increased cellular cytotoxicity [53]. However, zinc deficiency is linked to compromised selective intestinal permeability, which is observed in conditions like Crohn’s disease and ulcerative colitis, marked by structural and functional alterations of ZO-1 and OCLN [54]. A study in pigs indicated that oral supplementation with ZnO enhanced intestinal gene expression of these proteins [55]. Hence, it is plausible that ZnO contributes to protecting tight junctions, depending on its concentration and formulation. Our study also indicated that ZnO/HTs treatment significantly downregulated the gene expression of the gelatinase MMP9 enzyme, which is considered a direct contributor to damage incurred by intestinal epithelial tight junctions during inflammatory processes [56,57]. Elevated levels of MMP9 in chronic enteropathies can adversely affect tight junctions in the intestines, potentially exacerbating intestinal barrier damage and underlying inflammatory conditions. This underscores the importance of MMP9 in chronic enteropathies and suggests it could be a target for therapeutic and dietary interventions.

This study is the first to conduct an in vitro investigation of the combined effects of HTs and ZnO on critical biological functions of enterocytes. The compounds exhibited overlapping/additive properties in almost all of the analysed functional outcomes, with ZnO efficacy being positively influenced by their combined administration. To date, few studies have explored the combinatory effects of ZnO and tannins on gut health. Among them, in one of our previous studies, we found that in dogs, a combination of tannins and ZnO, along with other nutraceuticals, reduced symptoms associated with intestinal damage, improving stool consistency and frequency of evacuation, while Liu and colleagues demonstrated the efficacy of ZnO and tannins in preventing the onset of diarrhoea in weaning piglets [18,19]. From a cellular/biochemical perspective, the mechanisms behind this potentiation effect are yet to be fully elucidated. Tannins can form complexes with metals through the ortho-dihydroxyphenolic groups, modifying their structure–activity relationships [25,26,28]. A plausible effect could be that, as is the case for some polyphenols, HTs may enhance the intracellular uptake of ZnO, thereby amplifying its biological efficacy [58,59,60]. 

Using HTs to treat intestinal inflammatory conditions in animals could offer an ecological alternative, as they can be sourced from natural origins with sustainable extraction methods. Moreover, the environmental dispersion of HTs generally does not pose a public health hazard. In conclusion, our in vitro findings suggest that the combination of ZnO and HTs may hold the potential for enhancing the integrity and functionality of the intestinal barrier, particularly in contexts involving inflammation and oxidative stress, which are commonly associated with intestinal diseases. While our results are promising, further preclinical research is necessary to fully comprehend the mechanisms behind this beneficial interaction and to validate the practical applications of these compounds in clinical and nutritional settings.

## Figures and Tables

**Table 1:** Qualitative–quantitative analysis of the derivatives found in commercial chestnut dry extract. Percentages are calculated based on the concentration of individual derivatives in mmol/g.

Compound Class/Name	%
Gallotannins	26.65
Ellagitannins	73.35
Gallic acid	1.94
Castalagin + Vescalagin	28.30

## Data Availability

The datasets used and/or analyzed during the current study are avail-able from the corresponding author upon reasonable request.

## Supplementary Materials

The following supporting information can be downloaded at https://www.mdpi.com/article/10.3390/biom14060666/s1, Figure S1: MTT cytotoxicity assay; Figure S2: Validation of the inflammatory model.
