# Supplementary material for: Combination of Hydrolysable Tannins and Zinc Oxide on Enterocyte Functionality: In Vitro Insights
Source: Biomolecules. 2024 Jun 6;14(6):666. doi: 10.3390/biom14060666 (PMC11201419; doi:10.3390/biom14060666)
Supplement: Supplementary file 1 [file biomolecules-14-00666-s001.zip › biomolecules-2970647-supplementary.pdf]

5. Supplementary data

5.1 ZnO cytotoxicity

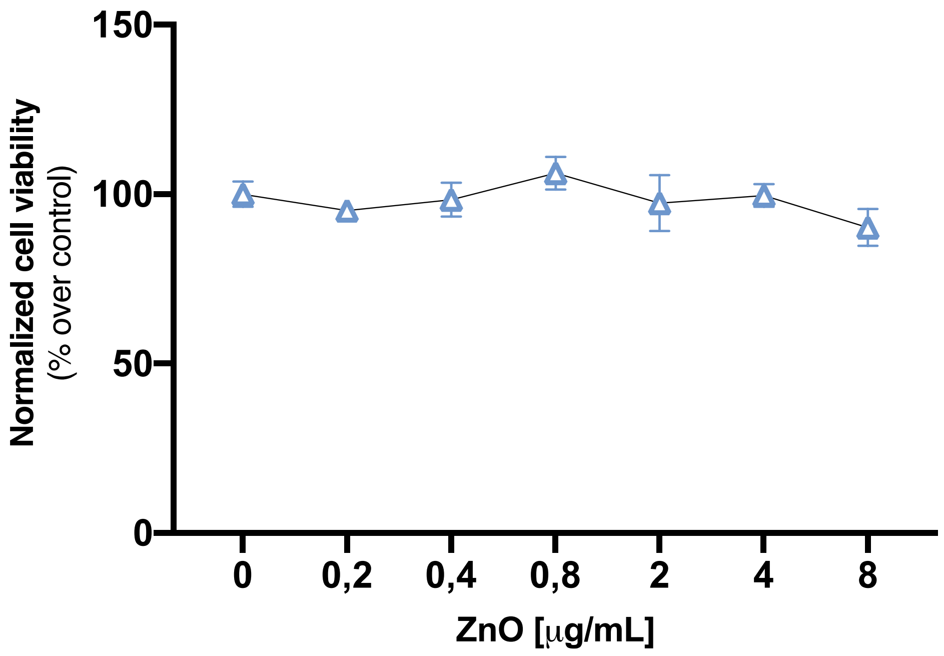

Figure S1. **MTT cytotoxicity assay.** MTT cytotoxicity assays on Caco-2 exposed to increasing concentrations of zinc oxide (ZnO) for 48 hours. Results are expressed as a percentage of untreated cells (i.e., 0 μg/mL) and presented as the mean ± standard deviation (SD) of three independent experiments.

5.2 Validation of the inflammatory model

Figure S2 illustrates the process of generating damage to the modelled barrier by establishing a co-culture between primary macrophages and differentiated Caco-2 monolayers. This damage is induced through the activation of macrophages (M1 polarization) with 100 ng/mL LPS and 10 ng/mL of INF-γ as described in the Material and Methods section. As a result, the transepithelial electrical resistance (TEER) values showed a reduction ranging from 20% to 50% compared to their initial values (when the co-culture was established). To provide additional supporting evidence, we established co-cultures with macrophages that were not induced to M1 polarization, hence in a state termed M0 or homeostatic. Notably, co-culture with the M0 phenotype did not result in a decline in TEER values (**Figure S2**).

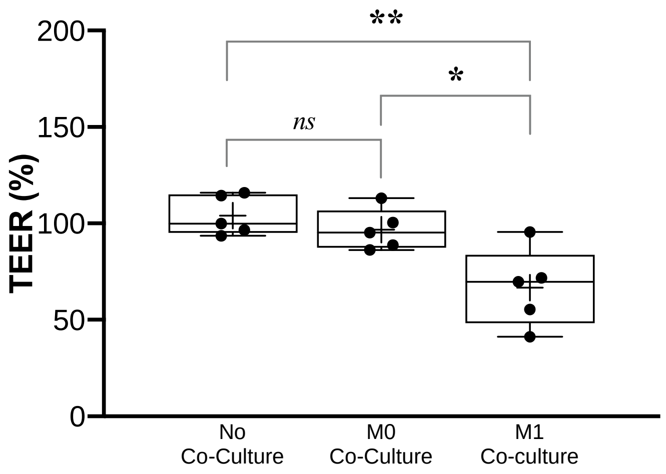

Figure S2. Validation of the inflammatory model. Transepithelial electrical resistance (TEER) measurements were taken 48 hours after establishing co-cultures involving differentiated human macrophages and Caco-2 monolayers grown on transwells. The graph presents TEER values at 48-hour expressed as a percentage of the values measured at the initial time point (T0). The three conditions represented are as follows: "No Co-Cultures," which involves only Caco-2 cells; "M0 Co-Culture," consisting of Caco-2 cells on transwells co-cultured with macrophages in a homeostatic state; and "M1 Co-Culture," where Caco-2 cells on transwells are co-cultured with macrophages polarized toward the proinflammatory phenotype. TEER values are expressed as a percentage of the initial value, calculated as (TEER at 48 hours from co-culture establishment /TEER at the co-culture establishment) × 100. The box plots feature a central line indicating the median, a cross for the mean value, and top and bottom edges for the third and first quartiles, respectively. The "whiskers" show data within 1.5xIQR (interquartile range), and black circles denote individual experimental data points. Statistical analysis was performed using a one-way ANOVA followed by post hoc multiple t-tests with Holm-Sidak's correction for multiple comparisons. Significance levels are denoted as: *P < 0.05, **P < 0.01, *ns*, not significant. Macrophages were isolated from n=5 different healthy donors.
